# Risk factors for macrophage activation syndrome in systemic juvenile idiopathic arthritis: a systematic review and meta-analysis

**DOI:** 10.3389/fped.2025.1695770

**Published:** 2025-12-05

**Authors:** Shihao Li, Xin Wu, Shuolan Jing, Xianglin Yang, Yuqing Zhang, Liqun Dong

**Affiliations:** 1 Division of Pediatric Pulmonology and Immunology, West China Second University Hospital, Sichuan University, Chengdu, China; 2 Key Laboratory of Birth Defects and Related Diseases of Women and Children, Ministry of Education, Sichuan University, Chengdu, China; 3 NHC Key Laboratory of Chronobiology, Sichuan University, Chengdu, Sichuan, China

**Keywords:** systemic juvenile idiopathic arthritis, macrophage activation syndrome, risk factors, case–control study, meta-analysis

## Abstract

**Background:**

Macrophage activation syndrome (MAS) is among the most life-threatening complications of systemic juvenile idiopathic arthritis (sJIA). Early identification of risk factors is critical for reducing mortality. However, existing evidence is markedly heterogeneous and lacks quantified, comparable, and clinically translatable conclusions.

**Methods:**

We systematically searched PubMed, Web of Science, EMBASE, and Chinese databases, including China national knowledge infrastructure (CNKI), Wanfang, VIP, and Chinese biomedical literature database (CBM), from inception to 1 July 2025, for case–control studies investigating risk factors for MAS in sJIA. Two investigators independently screened studies, extracted data, and assessed study quality. Meta-analyses were performed using RevMan 5.3 and Stata 16.0.

**Result:**

A total of 10 studies involving 1,936 patients were included, comprising 643 cases and 1,293 controls. Meta-analysis showed that central nervous system (CNS) involvement, hypofibrinogenemia, markedly elevated ferritin levels, and leukopenia were significantly associated with MAS in sJIA, with odds ratios (95% confidence intervals) of 4.30 (2.13–8.65), 4.03 (2.87–5.65), 8.28 (5.66–12.10), and 5.98 (2.80–12.75), respectively.

**Conclusion:**

CNS involvement, hypofibrinogenemia, hyperferritinemia, and leukopenia are core risk factors for MAS in patients with sJIA.

**Systematic Review Registration:**

PROSPERO CRD420251108082.

## 1 Introduction

Systemic juvenile idiopathic arthritis (sJIA) is one of the most common systemic inflammatory diseases in childhood. It is characterized by high fever, transient rash, hepatosplenomegaly, lymphadenopathy, and polyserositis. The pathogenesis of macrophage activation syndrome (MAS) involves complex interactions among hyperinflammation, cytokine release syndrome, and immune dysregulation. Some studies have emphasized the roles of cytokines such as IL-1β, IL-6, and IL-18 (1). Although secondary hemophagocytic lymphohistiocytosis (HLH) can occur in various diseases (2), MAS—as a subtype of secondary HLH—is relatively more common in patients with sJIA. Epidemiological data indicate that sJIA accounts for 10%–15% of all juvenile idiopathic arthritis cases (3). If inflammation is poorly controlled, the disease can rapidly progress to MAS, becoming its most severe and potentially fatal complication. MAS represents the rheumatic phenotype of secondary HLH (sHLH), whose pathophysiological hallmark is uncontrolled proliferation of cytotoxic T cells and macrophages, triggering a “cytokine storm” and leading to multiple organ failure. In 2016, the European League Against Rheumatism/American College of Rheumatology/Paediatric Rheumatology International Trials Organisation (EULAR/ACR/PRINTO) jointly issued the sJIA-MAS classification criteria, defining MAS as follows: in a patient with confirmed or suspected sJIA, serum ferritin >684 ng/mL plus at least two of the following—platelet count ≤181 × 10⁹/L, aspartate aminotransferase (AST) >48 U/L, triglycerides >156 mg/dL, or fibrinogen ≤360 mg/dL. However, in the original study, this standard shows only 73% sensitivity and is inadequate for detecting subclinical MAS in the context of biologic therapy or infection in a real-world setting (3).

The association between sJIA and MAS is not coincidental. On one hand, the IL-6-dependent inflammation intrinsic to sJIA can suppress the cytolytic function of NK cells and cytotoxic T lymphocytes (4), resulting in impaired immune clearance and creating conditions for persistent macrophage activation. On the other hand, recurrent infections, drug triggers, or abrupt increases in disease activity can serve as precipitating factors for MAS. Clinical reports indicate that the incidence of overt MAS is approximately 10%, whereas subclinical MAS may reach 30%–40% (5). Although MAS-related mortality has declined from 20%–60% in earlier periods to 8%–12% (6), it remains significantly higher than in patients with sJIA alone. Therefore, identifying quantifiable risk factors in the early stages of disease (7) and establishing stratified early warning models are crucial for the timely initiation of immunosuppressive or immunomodulatory therapy and for reducing mortality.

Nevertheless, current evidence shows marked heterogeneity: small sample sizes in single-center studies, inconsistent diagnostic criteria, racial differences, and complex treatment backgrounds make horizontal comparisons difficult. In view of this, the present study conducted a systematic review and meta-analysis integrating 10 case–control studies (*n* = 1,936 children) to quantify the effect sizes of core risk factors—central nervous system (CNS) involvement, hypofibrinogenemia, hyperferritinemia, and leukopenia—using evidence-based methods. The aim was to provide a robust evidence base for constructing a “clinical–laboratory–molecular” multidimensional early warning system and to lay the methodological foundation for subsequent prospective multicenter cohort studies.

## 2 Materials and methods

### 2.1 Search strategy and study selection

We systematically searched MEDLINE (via PubMed), Embase, Web of Science, the Cochrane Library, and four Chinese databases (CNKI, Wanfang, VIP, CBM) from inception to 1 July 2025, using MeSH terms and text words related to “systemic juvenile idiopathic arthritis,” “Still's disease,” and “macrophage activation syndrome,” combined with “risk factor(s)” (the complete search strategy is provided in Supplementary File S1). Reference lists of the included articles were manually screened. Only case–control or cohort studies comparing sJIA patients with and without MAS were eligible for inclusion. Articles in both English and Chinese were retrieved.

For inclusion in the present meta-analysis, original association studies were required to meet all of the following criteria:

Participants (P): pediatric patients (≤18 years) with a confirmed diagnosis of sJIA, regardless of sex. The case group consisted of patients with sJIA complicated by MAS, diagnosed according to internationally recognized criteria (e.g., 2005 Ravelli criteria or 2016 EULAR/ACR classification criteria). The control group comprised patients with sJIA without MAS.

Exclusion criteria: Studies were excluded if they met any of the following objective criteria: (1) incomplete raw data preventing extraction of 2 × 2 contingency tables for at least one risk factor; (2) failure to report diagnostic thresholds for MAS or laboratory parameters; (3) overlapping patient cohorts where >30% of participants were duplicated in another included study (verified by author/institution/cohort time period); or (4) case definitions that did not align with either the 2005 Ravelli or 2016 EULAR/ACR/PRINTO criteria.

Intervention/Exposure (I): potential risk factors for the development of MAS, including:

1. Clinical manifestations: duration of fever, degree of hepatosplenomegaly (palpable >3 cm below the costal margin), extent of lymphadenopathy, bleeding manifestations (purpura, mucosal bleeding), central nervous system (CNS) involvement (headache, seizures, altered consciousness)
2. Laboratory indicators: peripheral blood indices within 1 week prior to MAS onset (platelet count ≤262 × 10⁹/L, white blood cell (WBC) count ≤4.0 × 10⁹/L, reduced hemoglobin); liver function tests (AST >59 U/L, elevated alanine aminotransferase (ALT)); inflammatory and metabolic markers (marked increase in serum ferritin, fibrinogen ≤2.5 g/L, triglycerides ≥2.5 mmol/L, ferritin/erythrocyte sedimentation rate (ESR) ratio); immune parameters (reduced NK cell percentage and activity)
3. Potential triggers: active sJIA disease phase, infection (excluding Epstein–Barr virus), drug-induced factors

Comparators (C): sJIA patients without MAS, with their clinical characteristics and laboratory parameters serving as controls.

Outcomes (O): The primary outcome was the occurrence of MAS; secondary outcomes included MAS-related mortality, intensive care unit (ICU) admission, and other prognostic indicators.

Study Design (S): observational studies in the form of case–control or cohort designs providing evidence of baseline comparability. Case reports, reviews, uncontrolled case series, and commentary articles were excluded.

**Figure 1:**
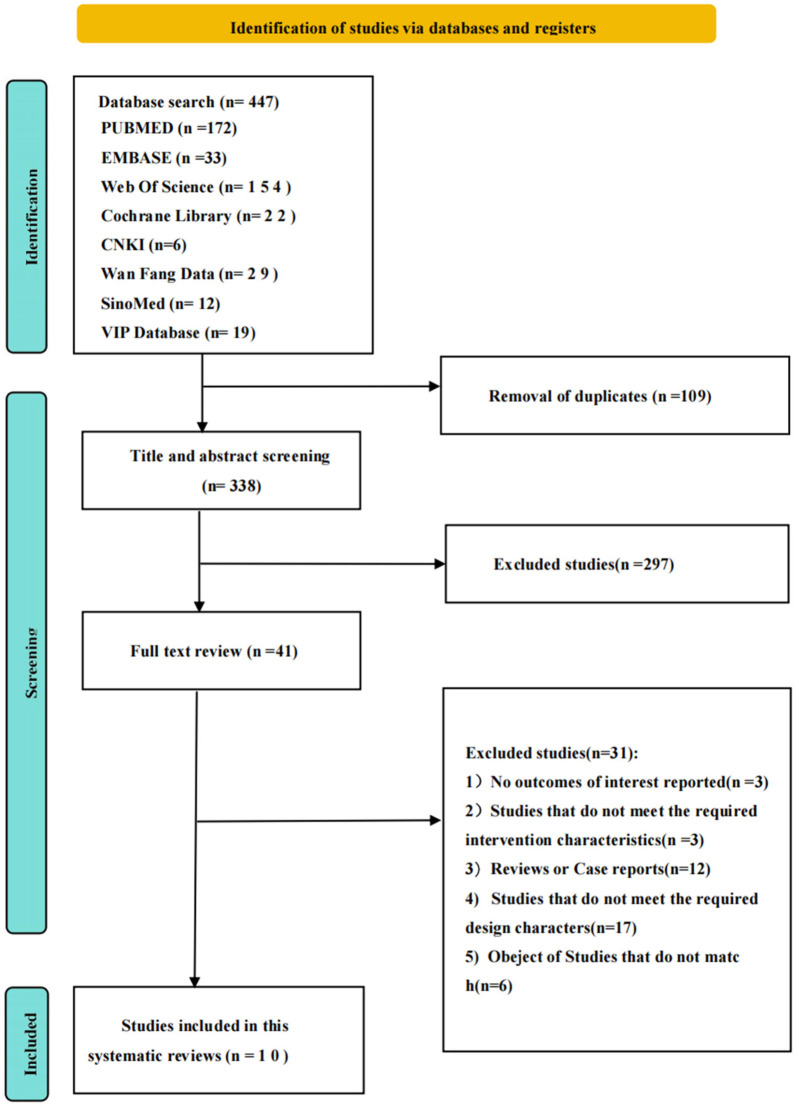
Flow diagram of all studies included in this meta-analysis; of the 447 studies identified via electronic and manual search, 10 were included.

The flow diagram of the study selection is shown in Figure 1.

### 2.2 Data extraction and quality assessment

A total of 10 studies were included in this meta-analysis. The following data were extracted from each publication: (a) first author, (b) year of publication, (c) country of origin, (d) sample size (cases/controls), and (e) diagnostic criteria. The Newcastle–Ottawa scale (NOS) for case–control studies was used to independently assess study quality by two authors (8). The NOS evaluates quality across three domains: selection, comparability, and exposure. A maximum of one point is awarded for each of the four selection criteria and each of the three exposure criteria, while up to two points can be awarded for comparability, yielding a maximum score of nine. Studies scoring ≤4 were deemed low quality, 4–6 moderate quality, and ≥7 high quality. Discrepancies between the two reviewers were resolved through joint re-evaluation of the original articles. For descriptive purposes, we summarized every variable that the original authors had evaluated, regardless of whether an association with MAS reached statistical significance. These items are listed in the “Variables examined in each study” column of Table 1; they were not pre-defined by the present review.

**Table 1:** The basic characteristics of the included studies.

First author	Sample size (cases/controls)	Country	Diagnostic criteria	Variables examined in each study	Literature quality score
Eloseily E. M. A. (10) 2019	524（262/262）	North America and Europe	2016 EULAR/ACR	Ferritin	★★★★★★
Guo, L. (11) 2021	149（27/122）	China	2016 EULAR/ACR	CNS involvement, ferritin, WBC	★★★★★★★★
Javaux, C. (12) 2021	78（9/69）	France	2016 EULAR/ACR	CNS involvement	★★★★★
Kostik, M. M. (13) 2015	58（18/40）	Russia	2005 年 Ravelli 标准	FIb, ferritin, WBC	★★★★★★
Shijie L. (14) 2017	210（70/140）	China	2016 EULAR/ACR	FIb, ferritin, WBC	★★★★★
Xiaohua T. (15) 2022	390 (141/249)	China	2016 EULAR/ACR	Fib, WBC	★★★★★★
Wenxiu X. (16) 2023	138（45/93）	China	2016 EULAR/ACR	Ferritin, Fib	★★★★★★★
Tingting Z. (17) 2021	107（36/71）	China	2016 EULAR/ACR	Fib	★★★★★★★
Rui Z. (18) 2022	107（36/71）	China	2016 EULAR/ACR	CNS involvement	★★★★★★★
Yaling Z. (19) 2011	195（8/187）	China	2005 年 Ravelli 标准	FIb, ferritin, WBC	★★★★★★★★

The column “Variables examined in each study” refers to parameters analyzed by the original authors; inclusion here does not indicate a significant association.

CNS, central nervous system; Fib, fibrinogen; WBC, white blood cell; EULAR/ACR, European League Against Rheumatism/American College of Rheumatology.

### 2.3 Statistical analyses

The pooled odds ratio (OR) with 95% CI was estimated using either a fixed-effect (Mantel–Haenszel method) or random-effect (DerSimonian–Laird method) model. Heterogeneity was quantified using the *I*^2^ statistic, where *I*^2^ < 30% indicated low heterogeneity, 30%–60% moderate, and >60% substantial heterogeneity (9). A fixed-effect model was applied when *I*^2^ < 50% and *p* ≥ 0.10 in the Cochran's *Q* test, indicating minimal between-study variance; otherwise, a random-effects model was used. This threshold aligns with Cochrane Handbook recommendations for meta-analyses of observational studies and was prespecified in our PROSPERO protocol. Publication bias was evaluated using funnel plots, Egger's test, and Begg's test.

Data synthesis and evaluation were conducted with RevMan 5.3 software. Sensitivity analyses were performed by excluding studies whose effect estimates deviated substantially from the 95% CI in the funnel plot and comparing the results to those obtained from analyses including all studies.

## 3 Results

### 3.1 Literature search results and basic characteristics of included studies

An initial search retrieved 447 relevant articles, including 27 in English and 10 in Chinese. According to the inclusion and exclusion criteria, 10 articles (10–19) were ultimately included, comprising 4 in English and 6 in Chinese, with a total of 1,936 participants—643 in the case group and 1,293 in the control group (Figure 1). All included studies were population-based case–control studies, and all participants were children under 16 years of age. Detailed characteristics of the included studies are presented in Table 1. All 10 studies were population-based case–control investigations performed in tertiary pediatric rheumatology centers. According to the NOS, none were of low quality: two studies received nine stars, four received eight stars, three received seven stars, and one received six stars (Supplementary Table S2). The main methodological strengths were a clear definition of sJIA and MAS cohorts, independent validation of exposure data from medical records, and comparable selection of cases and controls. The principal limitations across the body of evidence were retrospective design (7/10) and modest sample size (<100 subjects in 6/10 studies).

### 3.2 Meta-analysis of major risk factors

Based on the study content and the number of references available for each risk factor, four factors were selected for meta-analysis: CNS involvement, reduced fibrinogen, markedly elevated ferritin, and decreased WBC count.

#### 3.2.1 CNS involvement and sJIA-associated MAS

**Figure 2:**
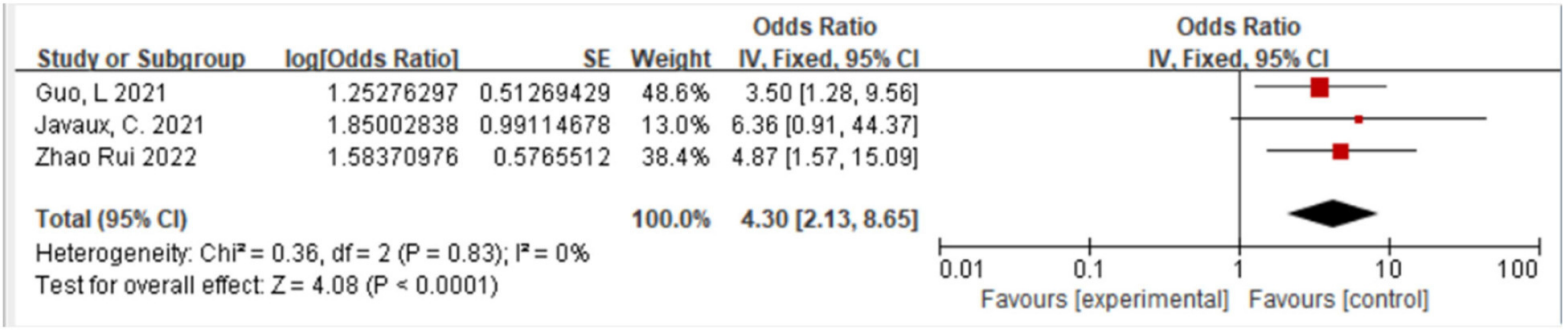
Forest plot of the meta-analysis of CNS involvement and sJIA-MAS.

A total of three studies (11, 12, 18) were included. Fixed-effect model meta-analysis indicated that CNS involvement showed a statistically significant difference between the case and control groups [OR = 4.30, 95% CI (2.13, 8.65), *P* < 0.00001], significantly increasing the risk of MAS. The proportion of CNS involvement was higher in the case group (Figure 2).

#### 3.2.2 Reduced fibrinogen and sJIA-associated MAS

**Figure 3:**
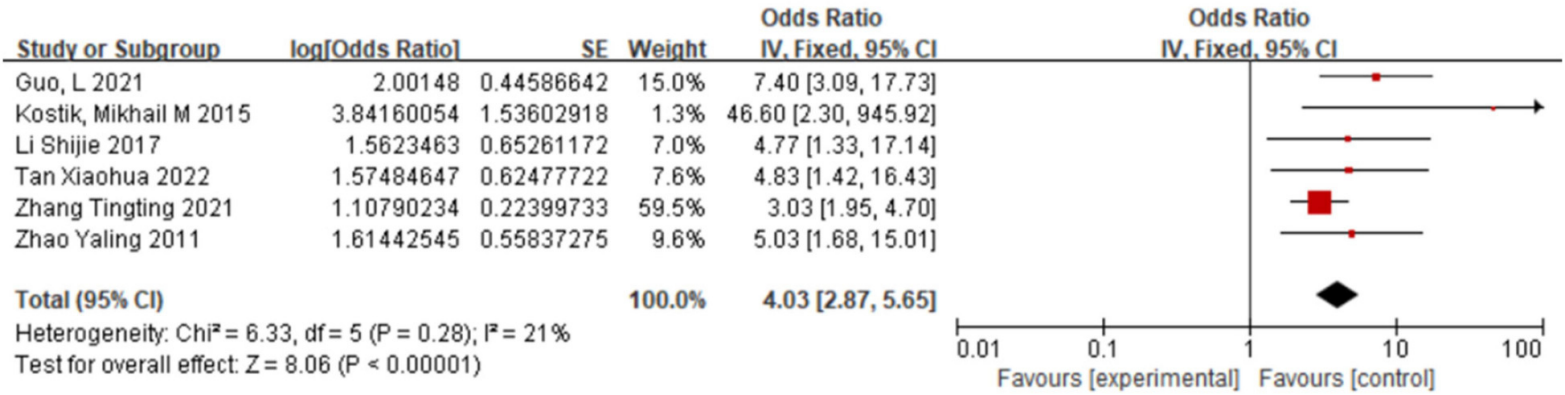
Forest plot of the meta-analysis of Fib and sJIA-MAS.

Seven studies (11, 13–15, 17, 19) were included. Fixed-effect model meta-analysis indicated that reduced fibrinogen was significantly more common in the case group than in the control group [OR = 4.03, 95% CI (2.87, 5.65), *P* < 0.00001], significantly increasing the risk of MAS (Figure 3).

#### 3.2.3 Markedly elevated ferritin and sJIA-associated MAS

**Figure 4:**
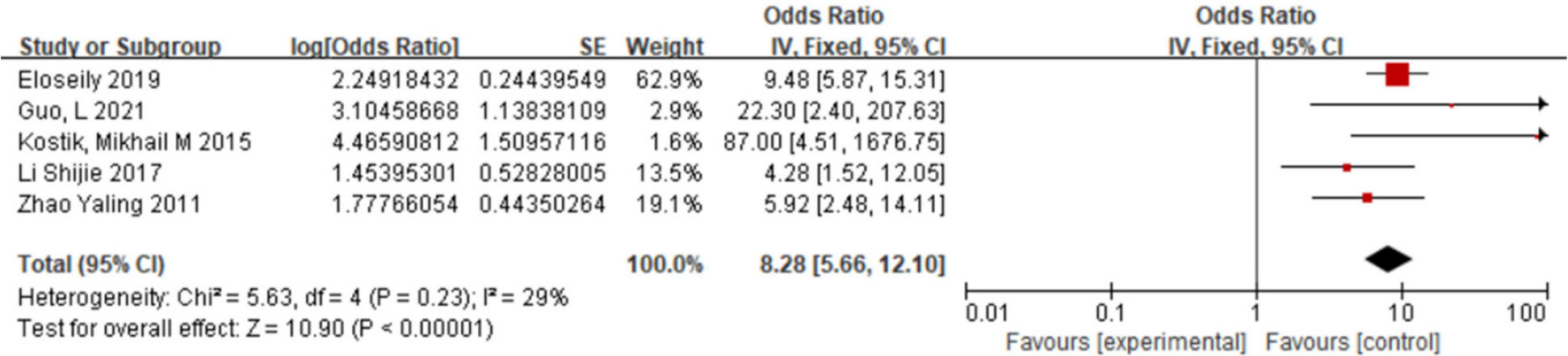
Forest plot of the meta-analysis of Ferritin and sJIA-MAS.

Five studies (10, 11, 13, 14, 19) were included. Fixed-effect model meta-analysis showed that markedly elevated ferritin was significantly more common in the case group than in the control group [OR = 8.28, 95% CI (5.66, 12.10), *P* < 0.00001], markedly increasing the risk of MAS (Figure 4).

#### 3.2.4 Decreased WBC count and sJIA-associated MAS

**Figure 5:**
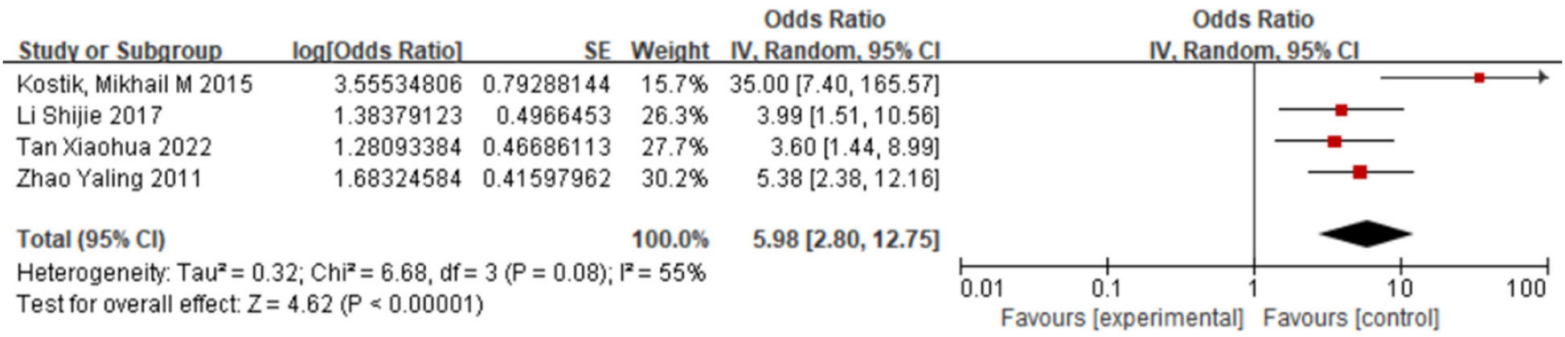
Forest plot of the meta-analysis of WBC and sJIA-MAS.

Four studies (13–15, 19) were included. Random-effect model meta-analysis indicated that decreased WBC count was significantly more common in the case group than in the control group [OR = 5.98, 95% CI (2.80, 12.75), *P* < 0.00001], significantly increasing the risk of MAS (Figure 5).

#### 3.2.5 Publication bias and sensitivity analyses

**Figure 6:**
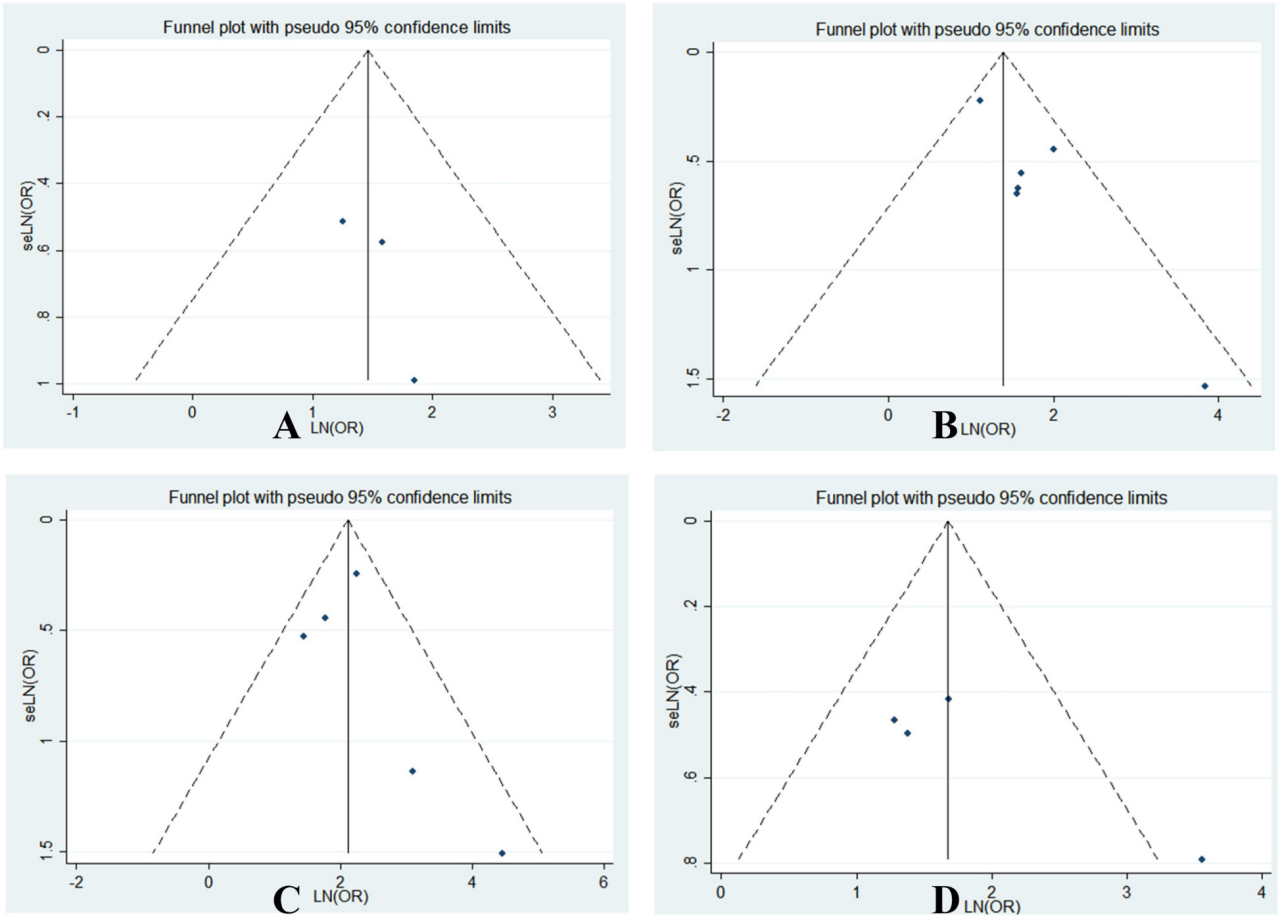
Funnel plot of the meta-analysis of CNS involvement **(A)**, Fib **(B)**, ferritin**(C)**, and WBC **(D)** of sJIA-MAS.

Funnel plots for CNS involvement, reduced fibrinogen, markedly elevated ferritin, and decreased WBC count in relation to sJIA-associated MAS were largely symmetrical (Figure 6). Symmetry tests yielded *P*-values of 0.380, 0.707, 0.566, and 0.146, respectively, indicating no significant publication bias. All *P*-values were >0.05, suggesting a low likelihood of publication bias in this meta-analysis.

## 4 Discussion

This study integrated 10 case–control investigations (*n* = 1,936) and, through meta-analysis, quantified four core risk factors for MAS complicating sJIA: CNS involvement (OR = 4.30, 95% CI 2.13–8.65), hypofibrinogenemia (OR = 4.03, 2.87–5.65), hyperferritinemia (OR = 8.28, 5.66–12.10), and leukopenia (OR = 5.98, 2.80–12.75). Tests for publication bias revealed no significant abnormalities, and sensitivity analyses indicated robust conclusions. These parameters provide clinicians with evidence-based early warning tools and lay a solid foundation for developing a multidimensional “clinical–laboratory–molecular” prediction model.

From a data-processing perspective, to ensure robustness of conclusions, we applied strict selection criteria to the original literature. First, studies reporting OR values >20 were excluded, as such extreme values typically arise from very small sample sizes or highly atypical single-center cases, which may overestimate effect sizes and introduce potential publication bias. Second, variables with OR values between 0.9 and 1.1 were excluded, as this range indicates a lack of discriminatory capacity between case and control groups. Finally, variables with OR <1 were removed, as they may reflect protective effects, which were beyond the scope of this study focused on risk factors. Sensitivity analyses demonstrated that these exclusions had minimal impact on the overall conclusions, suggesting that our findings are stable.

CNS involvement, manifesting as headache, altered consciousness, seizures, or cerebrospinal fluid abnormalities, indicates that the inflammatory storm has breached the blood–brain barrier. It is a hallmark sign of severe MAS and is significantly associated with poor prognosis (20, 21). Although the 2016 sJIA-MAS classification criteria do not explicitly include CNS manifestations, increasing evidence supports their consideration as “very high-risk” indicators to avoid missing the early intervention window. Ferritin is a sensitive marker of macrophage activation and iron metabolism dysregulation. A ferritin/ESR ratio >21.5 can effectively distinguish sJIA-MAS from active sJIA in most populations (sensitivity ≈ 82%, specificity ≈ 78%) (10). However, in the context of IL-6 inhibitor exposure (e.g., tocilizumab), ferritin elevations may be partially masked (22, 23), necessitating combination with IL-18, IFN-γ–axis biomarkers (e.g., CXCL9), and dynamic change rates to improve sensitivity (24). During active sJIA, fibrinogen levels are often elevated; a subsequent decline suggests a shift from “inflammation-induced hypercoagulability” to “phagocytic/consumptive coagulopathy,” representing one of the key laboratory signs of MAS (25). The 2016 classification criteria already include fibrinogen ≤360 mg/dL as one of four laboratory parameters. However, under IL-6 blockade therapy, the specificity of fibrinogen decline decreases, requiring combined assessment with ferritin, platelet count, and other markers (22, 23). In contrast to the leukocytosis often observed in active sJIA, MAS is characterized by significant leukopenia, reflecting immune dysregulation and macrophage overactivation (25). Studies have found that synchronous declines in white blood cells and platelets are strongly associated with MAS (26, 27), and the combination of ferritin, ferritin/ESR ratio, and platelet count is considered the strongest predictive panel (10, 24). It should be noted that agents such as tocilizumab can themselves induce leukopenia (28); therefore, interpretation must be made in the appropriate clinical context. The significant effects of ferritin and fibrinogen align with the high-inflammation, high-phagocytosis pathology of MAS (29). IL-6-dependent inflammation can suppress NK and cytotoxic T lymphocyte function, promoting sustained macrophage activation. Once inflammation breaches the blood–brain barrier, CNS involvement is more likely and signals a severe phenotype—consistent with prior reports of critical cases (29). The direction of our effect estimates also agrees with studies on adenosine deaminase 2 (ADA2), IL-18, and IFN-γ/IL-10 imbalance (24, 30, 31), suggesting that integrating molecular biomarkers with clinical and laboratory factors could enhance early detection capacity.

Despite providing statistically significant quantitative evidence, this study has several limitations. First, only 10 studies were included, predominantly from Eurasian populations, limiting ethnic representativeness and generalizability. Second, some studies had small sample sizes, leading to wide 95% CIs and reduced precision. Third, differences in MAS diagnostic criteria (2005 Ravelli vs. 2016 EULAR/ACR) may introduce bias, although heterogeneity tests did not reveal significant discrepancies (*I*^2^ < 50%). Fourth, none of the original studies reported biologic therapy history or infectious triggers (e.g., Epstein–Barr Virus (EBV)), precluding assessment of their modifying effects on MAS risk. Moreover, while meta-analysis addresses broad, average effects, it may overlook subgroup heterogeneity. For example, Guo et al. (11) reported that patients with hypotension had a higher ICU admission rate, suggesting that homogeneous statistical models may underestimate risk in specific clinical contexts. Additionally, most existing studies are retrospective, lacking longitudinal observation of risk factor trajectories, making it difficult to clarify the temporal sequence between “risk factors” and “disease progression.” At the molecular level, although evidence is growing, standardized testing protocols are lacking, and reproducibility and cutoff values for biomarkers such as ADA2 and IFN-γ/IL-10 require multicenter validation. Importantly, a single OR value cannot fully capture the dynamic pathophysiology of MAS. Recent research indicates that the risk factor spectrum is expanding. Lee et al. (32) first reported significantly elevated plasma adenosine deaminase 2 (ADA2) activity in sJIA-MAS patients (AUC = 0.939), regulated directly by IFN-γ and IL-18 (33), suggesting that the degree of monocyte/macrophage activation may serve as a dynamic monitoring window. Guo et al. (11) further found that the combination of sudden hypotension, serum IFN-γ > 17.1 pg/mL, and IL-10 > 7.8 pg/mL increased early MAS risk to OR = 142.5. Eloseily et al. (10) proposed the ferritin/ESR ratio >21.5 as a simple, accessible screening tool, especially in resource-limited settings. Integrating these biomarkers with the four core factors identified in our study could enable the construction of a three-tiered early warning framework: clinical (low blood pressure, CNS involvement), laboratory (dynamic changes in ferritin, fibrinogen, WBC, platelets), and molecular (ADA2, IFN-γ/IL-10 axis, IL-18 levels), facilitating the shift from population-level screening to individualized, precision risk prediction.

Although the 2024 EULAR/PReS position paper recommends the umbrella term “Still's disease,” we retained “sJIA” because all analyzed cohorts were confined to patients ≤16 years and were originally reported under this label.

## 5 Conclusion

Based on a meta-analysis of ten global case–control studies, this investigation is the first to establish—at an evidence-based level—that CNS involvement, hypofibrinogenemia, hyperferritinemia, and leukopenia constitute the four core risk factors for MAS in patients with sJIA. These findings provide robust quantitative support for constructing a three-dimensional early warning system integrating clinical, laboratory, and molecular parameters. Nevertheless, given the limitations of existing studies in terms of ethnic representation, uniformity of diagnostic criteria, and control of therapeutic background, large-scale, multicenter, prospective cohort studies with genetic stratification are urgently needed. Such studies should incorporate confounders including host genetics, drug exposure, and infectious triggers, thereby facilitating the transition from population-level risk prediction to individualized precision intervention, ultimately reducing MAS-related mortality and improving the long-term prognosis of children with sJIA.

## Data Availability

The original contributions presented in the study are included in the article/Supplementary Material; further inquiries can be directed to the corresponding author.
