# Supplementary material for: Risk factors for macrophage activation syndrome in systemic juvenile idiopathic arthritis: a systematic review and meta-analysis
Source: Front Pediatr. 2025 Dec 5;13:1695770. doi: 10.3389/fped.2025.1695770 (PMC12714893; doi:10.3389/fped.2025.1695770)
Supplement: Supplementary file 1 [file Table1.docx]

The complete search strategy is below.

("Arthritis, Juvenile"[Mesh] OR "juvenile idiopathic arthritis"[Title/Abstract] OR JIA[Title/Abstract] OR "juvenile rheumatoid arthritis"[Title/Abstract] OR "juvenile chronic arthritis"[Title/Abstract] OR "childhood arthritis"[Title/Abstract] OR "systemic juvenile arthritis"[Title/Abstract] OR "juvenile systemic arthritis"[Title/Abstract] OR sJIA[Title/Abstract] OR "Still's disease"[Title/Abstract] OR "juvenile-onset Still disease"[Title/Abstract])

AND

("Macrophage Activation Syndrome"[Mesh] OR "macrophage activation syndrome"[Title/Abstract] OR MAS[Title/Abstract] OR "secondary hemophagocytic lymphohistiocytosis"[Title/Abstract] OR "secondary HLH"[Title/Abstract])

AND

("Risk Factors"[Mesh] OR "risk factor"[Title/Abstract] OR predictor*[Title/Abstract] OR "case-control"[Title/Abstract] OR cohort[Title/Abstract])
